# A Bioactive Injectable Hydrogel Regulates Tumor Metastasis and Wound Healing for Melanoma via NIR‐Light Triggered Hyperthermia

**DOI:** 10.1002/advs.202402208

**Published:** 2024-05-05

**Authors:** Xueyi Liu, Meifang Shen, Tiejun Bing, Xinyun Zhang, Yifan Li, Qing Cai, Xiaoping Yang, Yingjie Yu

**Affiliations:** ^1^ State Key Laboratory of Organic‐Inorganic Composites Beijing Laboratory of Biomedical Materials College of Life Science and Technology Beijing University of Chemical Technology Beijing 100029 China; ^2^ Immunology and Oncology Center ICE Bioscience Beijing 100176 China

**Keywords:** cGAS‐STING pathway, injectable hydrogel, melanoma, mild hyperthermia, wound healing

## Abstract

Surgical resection remains the mainstream treatment for malignant melanoma. However, challenges in wound healing and residual tumor metastasis pose significant hurdles, resulting in high recurrence rates in patients. Herein, a bioactive injectable hydrogel (BG‐Mn^gel^) formed by crosslinking sodium alginate (SA) with manganese‐doped bioactive glass (BG‐Mn) is developed as a versatile platform for anti‐tumor immunotherapy and postoperative wound healing for melanoma. The incorporation of Mn^2+^ within bioactive glass (BG) can activate the cGAS‐STING immune pathway to elicit robust immune response for cancer immunotherapy. Furthermore, doping Mn^2+^ in BG endows system with excellent photothermal properties, hence facilitating STING activation and reversing the tumor immune‐suppressive microenvironment. BG exhibits favorable angiogenic capacity and tissue regenerative potential, and Mn^2+^ promotes cell migration in vitro. When combining BG‐Mn^gel^ with anti‐PD‐1 antibody (α‐PD‐1) for the treatment of malignant melanoma, it shows enhanced anti‐tumor immune response and long‐term immune memory response. Remarkably, BG‐Mn^gel^ can upregulate the expression of genes related to blood vessel formation and promote skin tissue regeneration when treating full‐thickness wounds. Overall, BG‐Mn^Gel^ serves as an effective adjuvant therapy to regulate tumor metastasis and wound healing for malignant melanoma.

## Graphical abstract

A bioactive injectable hydrogel (BG‐Mn^Gel^) is developed for anti‐tumor immunotherapy and wound healing of melanoma. Incorporation of Mn^2+^ in BG endows system with excellent photothermal properties, hence facilitating stimulator of interferon genes (STING) activation and reversing the tumor immune‐suppressive microenvironment. Furthermore, BG exhibits favorable angiogenic capacity and tissue regenerative potential, making it promising for the treatment of malignant melanoma.

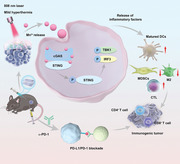

## 1 Introduction

Malignant melanoma is a highly metastatic and aggressive tumor that relies on surgery to remove superficial tumors and surrounding skin tissue.^[^ ^1^ ^]^ However, incomplete surgical resection and deeper tumor tissue lead to tumor recurrence or metastasis,^[^ ^2^ ^]^ resulting in a long and infection‐prone recovery period for postoperative skin defects.^[^ ^3^ ^]^ Therefore, a treatment that can effectively eliminate residual infiltrating tumor cells while accelerating wound healing is crucial for melanoma treatment.

In the past decade, immunotherapy has significantly changed the landscape of melanoma treatment. Immune checkpoint blockade (ICB), such as anti‐PD‐1 antibody (α‐PD‐1), can enhance the recognition between T cells and tumor cells, which reverses the immune suppression in the tumor microenvironment.^[^ ^4^ ^]^ However, its efficacy largely depends on the recruitment of tumor‐infiltrating lymphocytes (TILs).^[^ ^5^ ^]^ Photothermal therapy (PTT), which converts light energy to heat energy, has demonstrated great potential in reversing tumor‐suppressive environment.^[^ ^6^ ^]^ There is growing evidence that hyperthermia contributes to an increase in blood flow within the tumor microenvironment, facilitating the entry of immune cells such as TILs into the tumor, thereby enhancing their infiltration capabilities. Additionally, hyperthermia can directly affect the survival of tumor cells, making them more susceptible to recognition and elimination by the immune system.^[^ ^7^ ^]^

The cyclic GMP–AMP synthase (cGAS) – stimulator of interferon genes (STING) signaling pathway, as an important cytoplasmic DNA‐sensing pathway, takes part in regulating cancer immune response by triggering the production of type I interferons (IFN).^[^ ^8^ ^]^ Notably, manganese ion (Mn^2+^) has been shown to activate STING pathway.^[^ ^9^ ^]^ When activated by Mn^2+^, STING can facilitate the production of type I interferons and other pro‐inflammatory cytokines, boosting the immune system to detect and fight against cancer. Notably, this process can lead to increased infiltration of immune cells in tumor microenvironment, hence enhancing the recognition and elimination of tumor cells.

Beyond their robust anti‐tumor immune activation properties, both Mn^2+^ and mild hyperthermia play significant roles in tissue regeneration, synergistically promoting the recovery of damaged tissues by facilitating cell growth and reducing inflammation. Mn^2+^ plays a critical role in the regulation of integrin expression.^[^ ^10^ ^]^ Hyperthermia facilitates blood circulation by increasing the temperature of the damaged area. The enhanced blood flow delivers more oxygen and nutrients, supporting cellular repair and growth. The elevation of local tissue temperature can promote cell proliferation, and accelerate the tissue repair process.^[^ ^11^ ^]^ Collectively, the combination of Mn^2+^ and hyperthermia exhibit great promise in both anti‐tumor immunity and wound healing.^[^ ^12^ ^]^

**Scheme 1:**
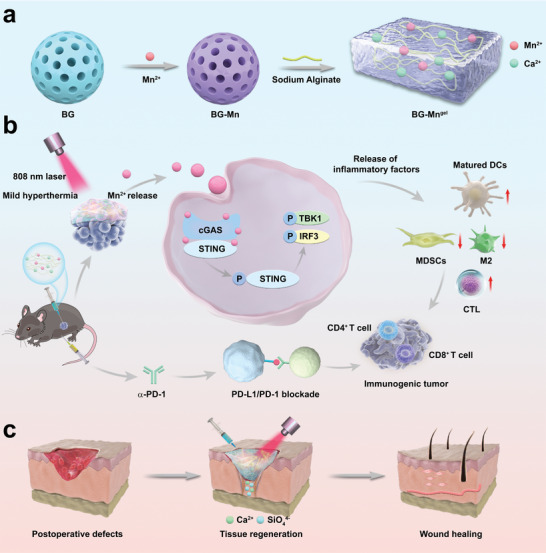
Schematic illustration showing a bioactive injectable hydrogel (BG‐Mn^gel^) for the regulation of tumor metastasis and wound healing for melanoma. a) Fabrication process of BG‐Mn^gel^ formed by crosslinking sodium alginate (SA) and ions (Mn^2+^ and Ca^2+^) released from BG‐Mn. b) Applications of BG‐Mn^gel^ for cancer immunotherapy and wound healing.

Herein, we synthesized Mn‐doped bioactive glass (BG‐Mn). On one hand, Mn^2+^ activates the STING pathway to elicit anti‐tumor response; on the other hand, the doping of Mn^2+^ endows the excellent photothermal properties to BG, which enhances the uptake of nanoparticles in cancer cells, thereby effectively improving anti‐tumor immune activation. Considering the potential of Mn^2+^ in stimulating the STING pathway, a platform enabling sustained release of Mn^2+^ in tumor tissue is highly desired. Hydrogel, crosslinked polymer networks infiltrated with water, serve as an ideal system to regulate tumor growth and wound healing for the treatment of melanoma.^[^ ^13^ ^]^ By blending BG‐Mn into sodium alginate (SA) solution, injectable composite hydrogels (BG‐Mn^gel^) were prepared, achieving in situ treatment of melanoma (**Scheme** **1**a).^[^ ^14^ ^]^ The near‐infrared (NIR) light‐mediated mild hyperthermia also enables accelerated release of Mn^2+^ in BG‐Mn^gel^, which facilitates immune activation and tissue regeneration at the tumor site (Scheme 1b). In vivo, we established a subcutaneous B16F10 tumor model in mice to evaluate the anti‐cancer effect of BG‐Mn^gel^. Through enhanced STING activation triggered by NIR light, BG‐Mn^gel^ can synergistically inhibit tumor growth. When combining with α‐PD‐1, BG‐Mn^gel^ activated long‐term anti‐tumor immune responses to prevent the recurrence of malignant melanoma.^[^ ^15^ ^]^ In in vivo wound healing model, BG‐Mn^gel^ promotes the angiogenic to achieve expeditious wound healing (Scheme 1c). Overall, BG‐Mn^gel^ serves as a versatile platform in regulation of tumor metastasis and wound healing, hence demonstrating a great potential in the treatment of malignant melanoma.^[^ ^16^ ^]^

## 2 Results and Discussion

### 2.1 Synthesis and Characterization of BG‐Mn

**Figure 1:**
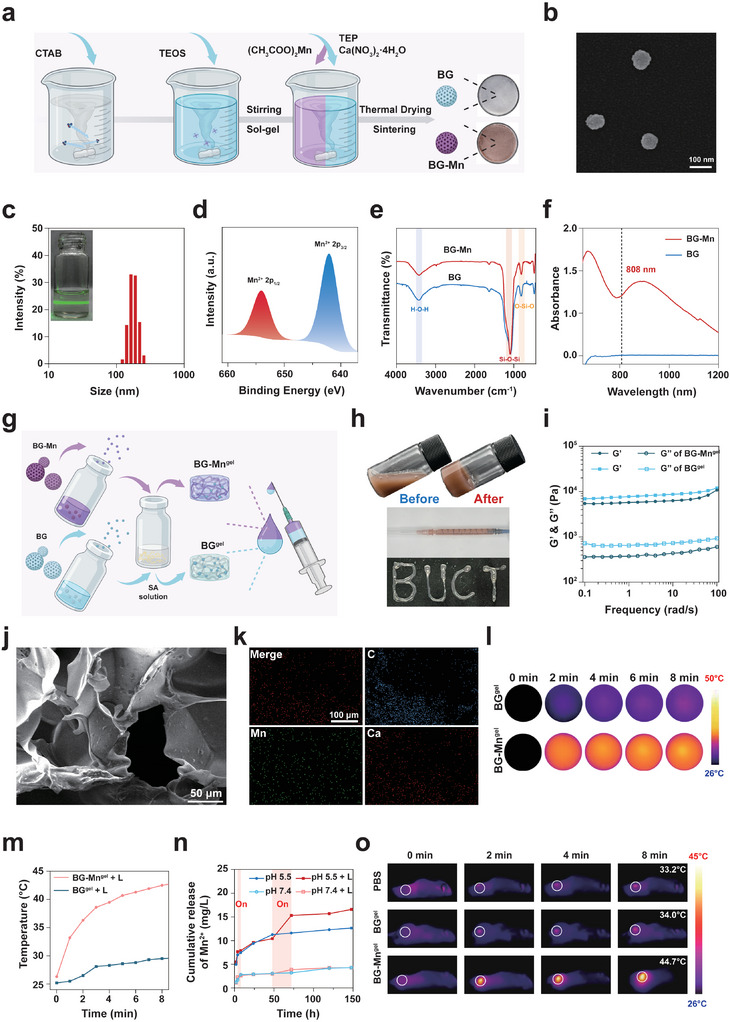
Preparation and characterization of BG‐Mn and BG‐Mn^gel^. a) A schematic showing the preparation and the optical images of BG and BG‐Mn. b) Representative SEM image of BG‐Mn. c) Size distribution of BG‐Mn detected by DLS. The inserted image represents the Tindal effect produced by BG‐Mn dispersed in PBS. d) X‐ray photoelectron spectroscopy (XPS) spectra of BG‐Mn. e) Fourier transform infrared spectroscopy (FT‐IR) spectra of BG and BG‐Mn. f) Ultraviolet‐visible (UV–vis) spectra of BG and BG‐Mn. g) Schematic illustration showing the preparation process of injectable BG‐Mn^gel^. h) Optical images of BG‐Mn^gel^ during gelation process and injectability of BG‐Mn^gel^. i) Rheological behavior analysis of BG^gel^ and BG‐Mn^gel^. j) SEM images showing the porous structure of BG‐Mn^gel^. k) The representative elemental mapping image of BG‐Mn^gel^ indicated the content of Mn, Ca, and C. l) Infrared thermal images of BG‐Mn^gel^ under 808 nm irradiation. m) Temperature profiles of BG^gel^ and BG‐Mn^gel^ under irradiation of 808 nm laser. n) Cumulative release profiles of Mn^2+^ from BG‐Mn^gel^ incubated in PBS (pH 5.5 or pH 7.4) with or without laser irradiation. o) In vivo infrared thermal images of the B16F10 melanoma‐bearing mice upon laser irradiation.

To achieve efficient loading of Mn^2+^, we synthesized Mn^2+^‐doped bioactive glass (BG‐Mn) using sol–gel method (**Figure** **1**a).^[^ ^17^ ^]^ Optical images showed that after incorporation of Mn^2+^, the powder color changes from white to brown (Figure 1a). The morphology of BG‐Mn was characterized through scanning electron microscopy (SEM), revealing that BG‐Mn exhibited a spherical morphology with uniform dispersion (Figure 1b). Dynamic light scattering (DLS) results showed the diameter distribution of BG‐Mn, which is consistent with SEM results (Figure 1c).

Subsequently, X‐ray photoelectron spectroscopy (XPS) was employed to characterize the element composition, revealing distinct signals of Ca, Si, P, and Mn in BG‐Mn (Figure S1, Supporting Information). Furthermore, the spectrum of Mn 2p signals exhibited strong peaks of Mn^2+^ 2p_1/2_ and Mn^2+^ 2p_3/2_, confirming the successful doping of Mn^2+^ in BG‐Mn (Figure 1d). Fourier‐transform infrared spectroscopy (FTIR) was utilized to investigate the composition of nanoparticles (Figure 1e). The results displayed absorption peaks at 3441 and 1100 cm^−1^, which correspond to H‐O‐H and Si‐O‐Si in BG and BG‐Mn. Figure 1f shows the UV–vis spectra of both nanoparticles. No absorption can be observed in BG. Conversely, with the incorporation of Mn^2+^, absorption peak can be observed at 808 nm in BG‐Mn.^[^ ^18^ ^]^ The photothermal properties of BG‐Mn were further characterized and the temperature of the solution under 808 nm laser irradiation gradually rose with increasing concentration of BG‐Mn suspension (Figure S2, Supporting Information). The photothermal conversion efficiency for BG‐Mn was calculated as 47.9%.^[^ ^19^ ^]^ Whereas the temperature of pure BG suspension remained nearly unchanged, demonstrating that the doping of Mn^2+^ effectively endowed BG with enhanced photothermal properties.

### 2.2 Preparation and Characterization of BG‐Mn^gel^

To achieve sustained Mn^2+^ release for topical treatment of melanoma, we continued to incorporate BG and BG‐Mn into sodium alginate (SA) solution to prepare BG and BG‐Mn contained hydrogel, respectively (denoted as BG^gel^ and BG‐Mn^gel^).^[^ ^20^ ^]^ Figure 1g shows the preparation process of BG‐Mn^gel^. The digital photographs show that upon mixing SA solution with BG or BG‐Mn solution, hydrogel was formed within 1 min in the presence of Ca^2+^ and Mn^2+^ released from BG and BG‐Mn, demonstrating the successful preparation of composite hydrogel (Figure 1h). The shape stability and controllability can be maintained after the injection. The rheology results showed that within the entire angular frequency range (0.1–100 rad s^−1^), the storage modulus (G′) of BG^gel^ and BG‐Mn^gel^ are consistently higher than the loss modulus (G″), indicative of a stable and solid‐like rheological behavior for both hydrogels (Figure 1i).

The morphology of the hydrogel was examined using SEM. Clearly, BG‐Mn was homogeneously dispersed in the porous structure of hydrogel (Figure 1j; Figure S3, Supporting Information). The elemental composition of BG‐Mn^gel^ was then analyzed by energy dispersive spectrometer (EDS), revealing a homogeneous distribution of C, Ca, and Mn across the hydrogel matrix (Figure 1k; Figure S4, Supporting Information). These results demonstrate the effective loading of BG‐Mn in the hydrogel. The compressive strength of BG^gel^ and BG‐Mn^gel^ was evaluated (Figure S5, Supporting Information). Given the remarkable photothermal characteristics of BG‐Mn, the photothermal responsiveness of the composite hydrogels was then assessed. Upon the irradiation of 808 nm laser (1 W·cm^−2^), the temperature of BG‐Mn^gel^ gradually increased to 43 °C (Figure 1l,m). As expected, the temperature of BG^gel^ does not increase obviously due to the absence of Mn^2+^. Excellent photothermal stability was observed on BG‐Mn^gel^ (Figure 1m). Additionally, the BG‐Mn^gel^ displayed superior photothermal stability (Figure S6, Supporting Information). This comprehensive evaluation highlights the enhanced photothermal efficacy and stability of BG‐Mn^gel^ owing to the incorporation of Mn^2+^.

Subsequently, we investigated the NIR‐light‐triggered Mn^2+^ release of BG‐Mn^gel^. As shown in Figure 1n, BG‐Mn^gel^ demonstrated a sustained release of Mn^2+^ in phosphate‐buffered saline (PBS) solutions at both pH 7.4 and pH 5.5. Conversely, both BG^gel^ and BG‐Mn^gel^ exhibited rapid release in acidic environments, possibly due to the pH sensitivity of SA, where ─COO^−^ converts to ‐COOH with reduced ionization. Notably, NIR‐induced mild hyperthermia accelerated the release rate of Mn^2+^ from BG‐Mn^gel^ at pH 5.5, hence demonstrating the light‐triggered Mn^2+^ release.

Having confirmed the excellent photothermal properties of BG‐Mn^gel^, we continued to study photoresponsiveness of hydrogel in B16F10 melanoma‐bearing mice upon laser irradiation. In vivo infrared thermal images showed that after intratumoral injection of BG‐Mn^gel^, the temperature at the tumor site of mouse rapidly increased to 44.7 °C in 8 min. Conversely, only negligible temperature can be observed at the tumor site of mice treated with PBS and BG^gel^ (Figure 1o). Together, these results indicate that the BG‐Mn^gel^ can achieve sustained release of Mn^2+^ and controlled accelerated release via NIR light induced‐mild hyperthermia.

### 2.3 In Vitro Biocompatibility Study

Biocompatibility is a prerequisite for biomedical applications. Therefore, we investigated the effects of BG‐Mn^gel^ on the proliferation and migration of fibroblasts in vitro. Live/dead staining demonstrated that L929 fibroblasts maintained good cell viability during the 5‐day co‐culture with BG^gel^ and BG‐Mn^gel^ (Figure S7, Supporting Information). These findings indicate that BG‐Mn^gel^ exhibits excellent biocompatibility, making it suitable for in vivo applications.

The long‐term absence of reparative cells due to skin lesions after skin cancer treatment severely impairs wound healing.^[^ ^21^ ^]^ Rapid cell migration is necessary for wound repair. Therefore, we investigated the effects of bioactive hydrogels on the migration of L929 cells through a scratch assay.^[^ ^22^ ^]^ As shown in Figures S8 and S9 (Supporting Information), the PBS group exhibited minimal cell migration into the scratched area, while both the BG^gel^ and BG‐Mn^gel^ groups showed a significant number of cells migrating into the scratched area. After 24 h, the migration area in the BG‐Mn^gel^ and BG‐Mn^gel^ groups was much larger than that in the Blank group. Overall, BG‐Mn^gel^ exhibited excellent biocompatibility.

### 2.4 Intracellular Uptake and STING Activation in Cancer Cells

**Figure 2:**
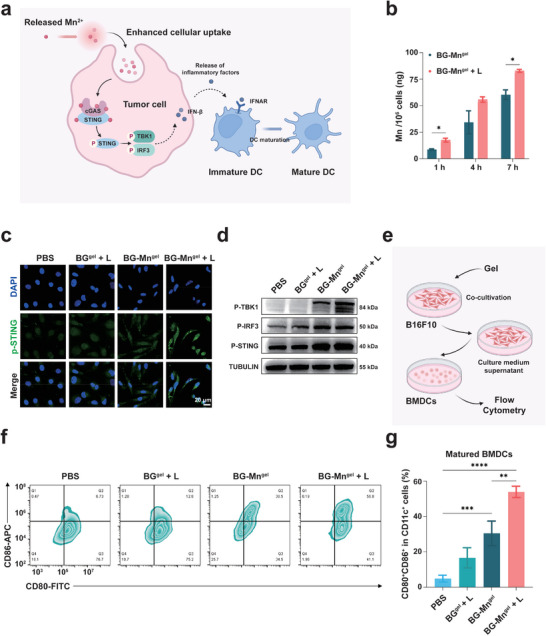
BG‐Mn^gel^ potentiates cGAS‐STING activation enhanced by mild hyperthermia in vitro. a) Schematic showing the biological mechanism of released Mn^2+^ in activating the cGAS‐STING pathway in tumor cells with mild hyperthermia treatment. b) Intracellular uptake of Mn in B16F10 cells treated with BG‐Mn^gel^. c) The CLSM images showed the expression of p‐STING in B16F10 cells upon various treatments. d) Western blot analysis showing the expression levels of STING pathways related proteins in B16F10 cells upon various treatments. e) Schematic illustration showing the experimental process for evaluation of bone marrow‐derived dendritic cells (BMDCs) maturation by flow cytometry. f) Flow cytometric profiles and g) the corresponding semi‐quantitative analysis of activated DC cells after incubation with B16F10 cells upon various treatments. Data represent mean ± standard deviation (SD) from *n* independent experiments (*n* = 3). Statistical significances between every two groups were calculated via one‐way ANOVA. ^**^ *p* < 0.01, ^***^ *p* < 0.001, ^s^ *p* < 0.0001.

As depicted in **Figure** **2**a, the activation of the stimulator of interferon genes (STING) pathway in tumor cells is closely related to anti‐tumor immunity.^[^ ^23^ ^]^ First, we investigated the cellular internalization of Mn^2+^ in B16F10 cells using atomic absorption spectrometry (AAS). The results showed that the accumulation of Mn^2+^ increased gradually with the treatment time of BG‐Mn^gel^ (Figure 2b). Moreover, upon 808 nm laser irradiation, the heat generated from BG‐Mn^gel^ not only promoted the release of Mn^2+^ from the hydrogel but also significantly enhanced the cellular uptake of Mn^2+^. At 7 h post‐treatment, the Mn^2+^ accumulation in B16F10 cells treated with BG‐Mn^gel^ + L reached 82.86 ng/10^6^ cells, which is 1.37 times higher than that with BG‐ Mn^gel^, hence demonstrating the efficient Mn^2+^ cellular internalization with NIR irradiation.

The presence of Mn^2+^ enhances the activation of the cyclic GMP‐AMP synthase (cGAS)‐STING pathway, from promoting the production of cyclic GMP‐AMP (cGAMP) to enhancing the affinity between cGAMP and STING.^[^ ^24^ ^]^ Therefore, we assessed the capacity of BG‐Mn^gel^ in STING activation in vitro. The immunofluorescence staining was carried out to visualize phosphorylated STING (p‐STING) in B16F10 cells, providing insight into the mechanistic efficacy of BG‐Mn^gel^ in modulating STING activation.^[^ ^25^ ^]^ In Figure 2c, confocal laser scanning microscopy (CLSM) images showed stronger green fluorescence intensity in the nuclei of cells treated with BG‐Mn^gel^ + L, indicating that the combination of BG‐Mn^gel^ and mild hyperthermia can effectively induce STING activation. Quantification of the intracellular fluorescence intensity further revealed that the green fluorescence intensity in cells treated with BG‐Mn^gel^ + L was 1.38 times higher than that with BG‐Mn^gel^ (Figure S10, Supporting Information). Subsequently, we examined the expression of STING pathway‐related proteins using Western blot. As depicted in Figure 2d, the expression of phosphorylated TBK1 (P‐TBK1), phosphorylated IRF3 (P‐IRF3), and phosphorylated STING (P‐STING) increased in cells treated with BG‐Mn^gel^ and BG‐Mn^gel^ + L. Notably, cells treated with BG‐Mn^gel^ + L exhibited the most significant upregulation of protein levels, surpassing those treated with BG‐Mn^gel^ alone, consistent with the aforementioned CLSM images. Together, these results collectively demonstrate that BG‐Mn^gel^ can effectively activate the STING pathway, and mild hyperthermia can further enhance this process.

The maturation and activation of antigen‐presenting cells play a crucial role in STING‐mediated anti‐tumor immunity. To validate the ability of BG‐Mn^gel^ in augmenting the maturation of dendritic cells (DCs) in vitro, we co‐cultured B16F10 cells treated with different conditions with mouse bone marrow‐derived dendritic cells (BMDCs) (Figure 2e).^[^ ^26^ ^]^ The maturation level of BMDCs was detected using flow cytometry. As depicted in Figure 2f, BG‐Mn^gel^ + L induced the highest proportion of mature BMDCs (53.93%), which was 11.0, 3.2, and 1.7 times higher than those treated with PBS, BG^gel^ + L, and BG‐Mn^gel^, respectively (Figure 2g). In summary, the above results indicate that BG‐Mn^gel^ + L can enhance cellular uptake of Mn^2+^ to activate the STING pathway, induce phosphorylation of IRF3, TBK1, and STING proteins, and ultimately promote the maturation of BMDCs.

### 2.5 RNA‐Sequencing Analysis

**Figure 3:**
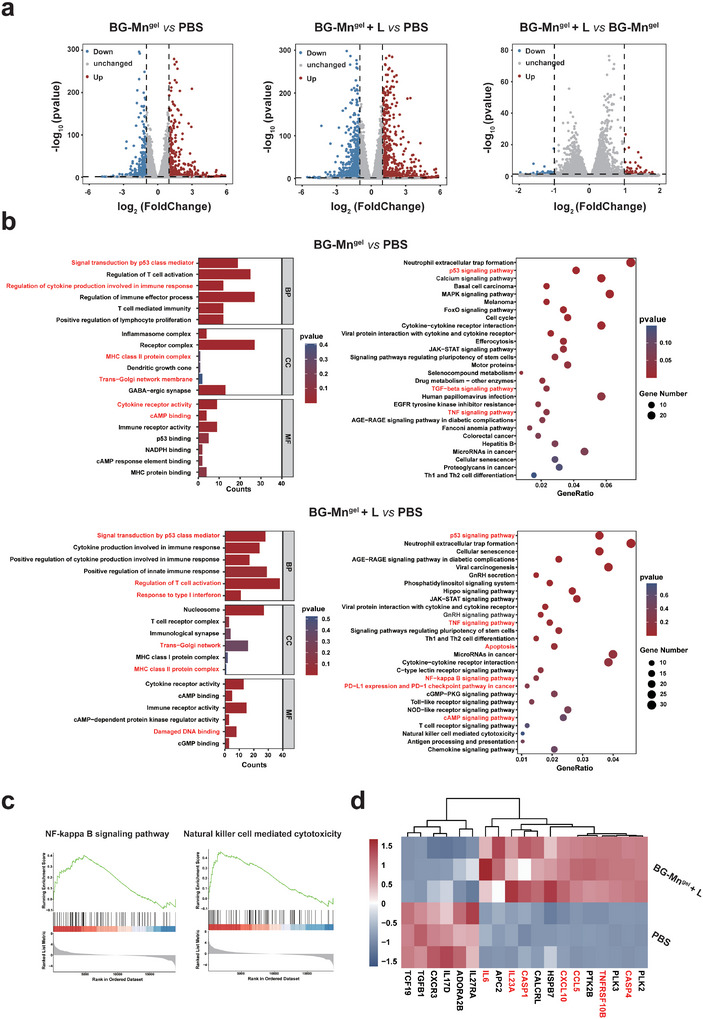
Transcription analysis of B16F10 cells by RNA‐seq after various treatments. a) Volcano plots displayed the differentially expressed genes between cells with different treatments. b) Gene Ontology (GO) and Kyoto Encyclopedia of Genes and Genomes (KEGG) analyses of differentially expressed genes between cells treated with BG‐Mn^gel^ and PBS, BG‐Mn^gel^ + L and PBS. c) GSEA analysis showed the genes sets of NF‐kappa B signaling pathway and natural killer cell mediated pathway. d) Heat‐map of gene expression in cells treated with BG‐Mn^gel^ + L and PBS.

To investigate the anticancer mechanism of BG‐Mn^gel^, we conducted genome‐wide RNA‐seq analysis on B16F10 cells treated with different groups.^[^ ^23^ ^]^ **Figure** **3**a illustrates the gene correlations within each group of cells. Notably, 798 genes were upregulated, and 912 genes were down‐regulated in cells treated with BG‐Mn^gel^ + L compared to those treated with PBS. Furthermore, Kyoto Encyclopedia of Genes and Genomes (KEGG) and Gene Ontology (GO) enrichment analysis showed that BG‐Mn^gel^ and BG‐Mn^gel^ + L predominantly affected genes in the p53, IFN‐I, and TNF signaling pathway (Figure 3b). The TNF signaling pathway refers to the biological pathway triggered by TNF, which is a cytokine involved in systemic inflammation. The TNF signaling pathway involves the activation of NF‐kB and MAPK, which are important for inflammatory responses and cell survival. This results have also been validated in KEGG enrichment analysis. This is due to BG‐Mn^gel^ and hyperthermia inducing STING activation, where STING, through its interactions with the IFN‐I, TNF, and NF‐kB signaling pathways, collaboratively regulates anti‐tumor immunity.^[^ ^27^ ^]^ These results collectively indicate that BG‐Mn^gel^ +L successfully activates STING pathway, downstream immune cells, and signaling pathways related to anti‐tumor immunity in vitro. Interestingly, we observed an increase in PD‐L1 expression and the emergence of the PD‐L1 and PD‐1 checkpoint pathway in BG‐Mn^gel^ + L. Therefore, in subsequent in vivo experiments, the use of a‐PD‐1 in combination could elicit robust immune response.

Previous studies have indicated that Mn^2+^ can inhibit tumor growth via STING‐activated anti‐tumor immunity. To investigate whether BG‐Mn^gel^ + L can affect the immune pathways activated by STING, we conducted a Gene Set Enrichment Analysis (GSEA). The results display pathways related to STING activation in B16F10 cells, such as the NF‐ kB signaling pathway amd NK‐cell‐mediated cytotoxicity (Figure 3c).^[^ ^28^ ^]^ This indicated the anti‐tumor immune response induced by STING activation.

To further explore whether BG‐Mn^gel^ + L could reverse the immune suppressive tumor microenvironment, the heat‐map of gene expression in cells was presented. The results revealed that genes such as CXCL10 and CCL5 were up‐regulated in cells treated with BG‐Mn^gel^ + L (Figure 3d). At the same time, the expression of immune‐stimulating factors, such as IL6 and IL23A, was up‐regulated in the cells treated with BG‐Mn^gel^ + L. Therefore, BG‐Mn^gel^ + L can reverse the immunosuppressive microenvironment by stimulating the release of immune activation factors and proinflammatory factors. In summary, BG‐Mn^gel^ + L can promote STING activation, inducing a series of anti‐tumor immune responses, enhancing the expression and recruitment of immune stimulatory factors, and ultimately reverse the immunosuppressive microenvironment to achieve enhanced immune response.

### 2.6 In Vivo Immune Activation Evaluation

**Figure 4:**
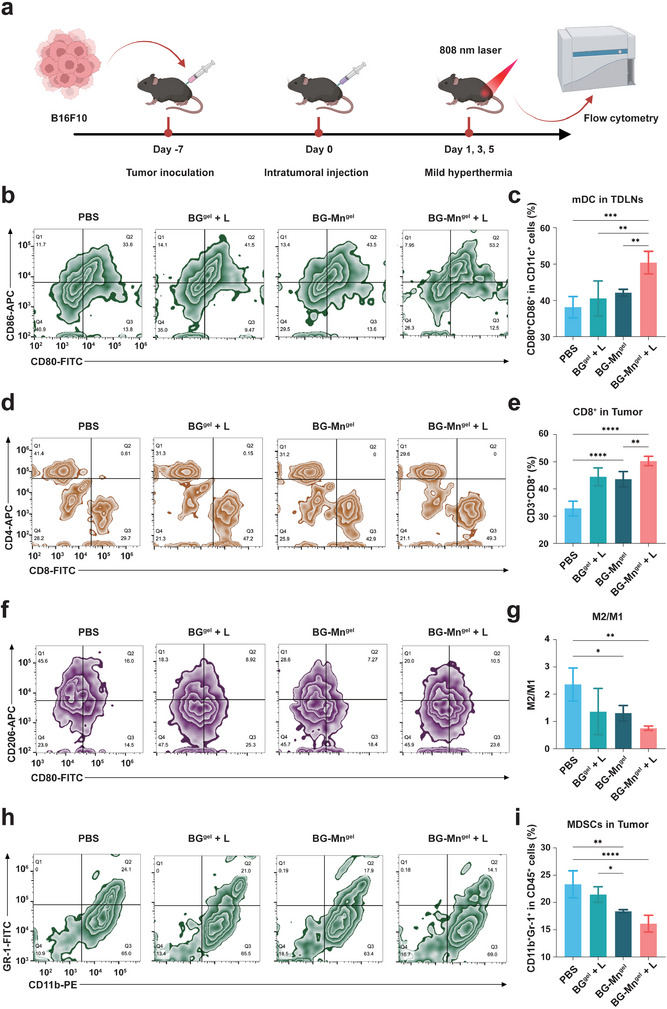
In vivo immune activation of BG‐Mn^gel^. a) Schematic illustration showing the evaluation of the immune response after various treatments on B16F10 tumor‐bearing C57BL/6 mice. b) Flow cytometric analysis and c) the corresponding quantitative analysis of mDCs in TDLNs of mice with different treatments. d) Flow cytometric analysis of CD4^+^ and CD8^+^ cells gating on CD3^+^ cells in the tumor tissues and e) the corresponding percentages of populations of CD3^+^ and CD8^+^ T cells in mice with various treatments. f) Flow cytometric analysis of CD206 and CD80 cells in the tumor tissues and g) the corresponding percentages of populations of M2/M1 cells in each group. h) Flow cytometric analysis and i) the corresponding quantification of MDSCs infiltrated in tumor tissues. Data represent mean ± standard deviation (SD) from *n* independent experiments (*n* = 5). Statistical significances between every two groups were calculated via one‐way ANOVA. ^**^ *p* < 0.01, ^***^ *p* < 0.001, ^****^ *p* < 0.0001.

Having confirmed the activation of cGAS‐STING pathway, we continued to evaluate in vivo immune activation of BG‐Mn^gel^. Notably, STING activation promotes the maturation of DCs, tumor‐specific antigen presentation, enhances the activation of CD8^+^ T cells, and increases the generation of memory CD8+ T cells.^[^ ^29^ ^]^ Furthermore, mild hyperthermia can reshape the tumor immune‐suppressive microenvironment and is considered a key factor in generating a favorable tumor immune environment.^[^ ^30^ ^]^ To investigate the synergistic effects of Mn^2+^ and mild hyperthermia on immune activation in vivo, we applied BG‐Mn^gel^ to melanoma tumor‐bearing mice. Tumor and lymphoid tissues were extracted from mice treated with different conditions, and flow cytometry was used to analyze the populations of immune cells (**Figure** **4**a).

The maturation of DCs is crucial for T cell activation and anti‐tumor immunity. Therefore, we quantitatively assessed the presence of mature DCs (mDCs, CD11c^+^CD80^+^CD86^+^) in tumor‐draining lymph nodes (TDLNs) using flow cytometry. The results showed that the proportion of mDCs in the BG‐Mn^gel^ + L group was as high as 50.4%, which was 1.19 times higher than that in the BG‐Mn^gel^ group (Figure 4b,c; Figure S11, Supporting Information). Furthermore, the number of cytotoxic T lymphocytes (CD8^+^ T cells, CD3^+^CD8^+^) in tumor tissues was 50.26% with BG‐Mn^gel^ + L treatment, which was 1.15 times higher than that of the BG‐Mn^gel^ group (Figure 4d,e; Figure S12, Supporting Information). M1 macrophages (M1, FA/80^+^CD80^+^CD206^−^) play a crucial role in immune surveillance by secreting pro‐inflammatory cytokines and chemokines while actively presenting antigens to induce positive immune responses. However, M2 macrophages (M2, F4/80^+^CD80^−^CD206^+^) with immunosuppressive characteristics can inhibit the activation and proliferation of effector T cells, leading to tumor immune evasion. Flow cytometry results showed that the ratio of M2 to M1 cells in tumor tissues treated with BG‐Mn^gel^ + L was ≈0.75:1, which is 0.58 times lower than that of BG‐Mn^gel^ (Figure 4f,g; Figure S13, Supporting Information). Furthermore, the proportion of myeloid‐derived suppressor cells (MDSCs) in tumor tissues of mice treated with BG‐Mn^gel^ + L group was markedly reduced (Figure 4h,i; Figure S14, Supporting Information), corresponding to the M2/M1 ratio in that group. This suggests that BG‐Mn^gel^ + L can significantly improve the tumor immune‐suppressive microenvironment, leading to increased proliferation of immune‐activated cells and suppression of the growth of immune‐suppressive cells at the tumor site.^[^ ^31^ ^]^

### 2.7 Combination Therapy of BG‐Mn^gel^ and a‐PD‐1 for Immune Activation In Vivo

**Figure 5:**
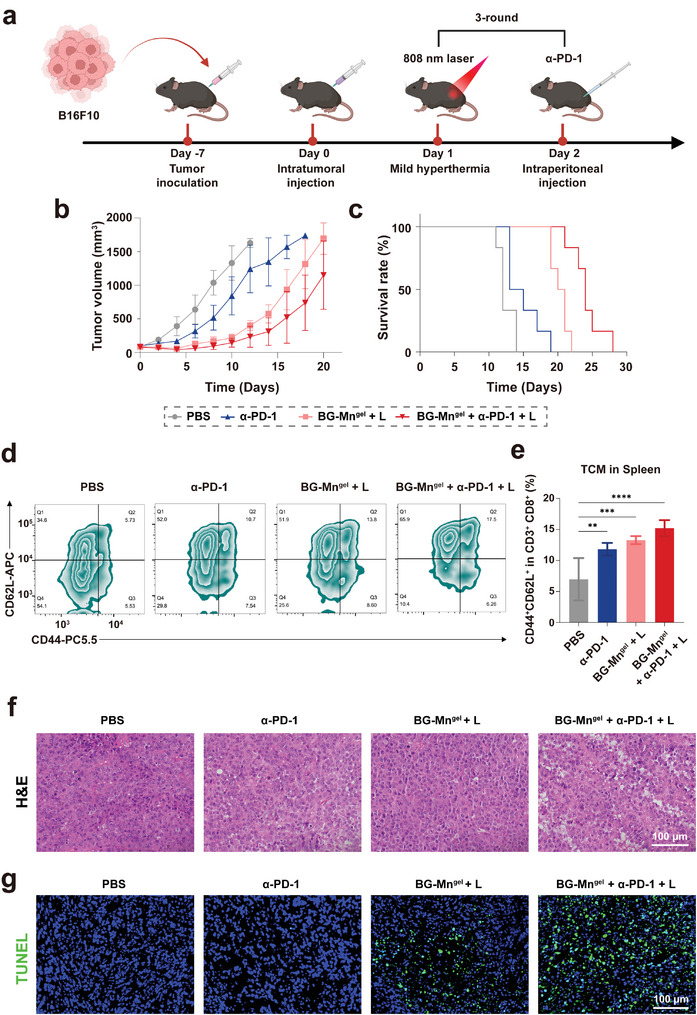
Combination therapy of α‐PD‐L1 and BG‐Mn^gel^ treatment activate long‐term immune effects. a) Schematic illustration showing the evaluation of the long‐term immune response after combination therapy in B16F10 tumor‐bearing C57BL/6 mice. b) Tumor growth inhibition curves after various treatments in mice. c) Survival rate of mice with various treatments. d) Flow cytometric analysis of CD62L and CD44 cells in the spleen of mice with various treatments and e) the corresponding percentages of population of memory CD8^+^ T cells in each group. f) H&E staining of residual tumors after the various treatments. g) TUNEL staining of representative tumor tissues after various treatments. Data represent mean ± standard deviation (SD) from *n* independent experiments (*n* = 5). Statistical significances between every two groups were calculated via one‐way ANOVA. ^**^ *p* < 0.01, ^***^ *p* < 0.001, ^****^ *p* < 0.0001.

Immune checkpoint blockade (ICB), such as anti‐PD‐1 antibody (α‐PD‐1), aims to reverse immune suppression in the tumor microenvironment. We continued to investigate the synergistic effect of combining BG‐Mn^gel^ + L with α‐PD‐1 (**Figure** **5**a).^[^ ^32^ ^]^ In Figure 5b, the tumor growth curve showed that BG‐Mn^gel^ + α‐PD‐1 + L group can significantly suppress tumor growth compared to other groups. Moreover, the survival time of mice in the BG‐Mn^gel^ + α‐PD‐1 + L group was significantly prolonged (Figure 5c).

Therefore, we further investigated the immune memory effect in mice by measuring the proportion of central memory T (T_CM_) cells in tumor tissues. T_CM_ cells correspond to antigen recognition, proliferation, and differentiation into effector cells.^[^ ^33^ ^]^ As shown in Figure 5d,e, the proportion of T_CM_ cells significantly increased in the BG‐Mn^gel^ + α‐PD‐1 + L group. The tumor tissue of mice after various treatments was further assessed through histological examination and immunofluorescence staining.^[^ ^34^ ^]^ Hematoxylin and eosin (H&E) staining revealed significant damage in tumor section of mice treated with BG‐Mn^gel^ + α‐PD‐1 + L (Figure 5f). Furthermore, terminal deoxynucleotidyl transferase dUTP nick end labeling (TUNEL) staining of tumor sections showed that the highest apoptosis amount of dead cells in BG‐Mn^gel^ + α‐PD‐1 + L group (Figure 5g). These results indicate that the combination of ICB and BG‐Mn^gel^ can induce robust T cell memory effects, hence activating long‐term anti‐tumor immune responses.

### 2.8 In Vivo Wound Healing Evaluation

**Figure 6:**
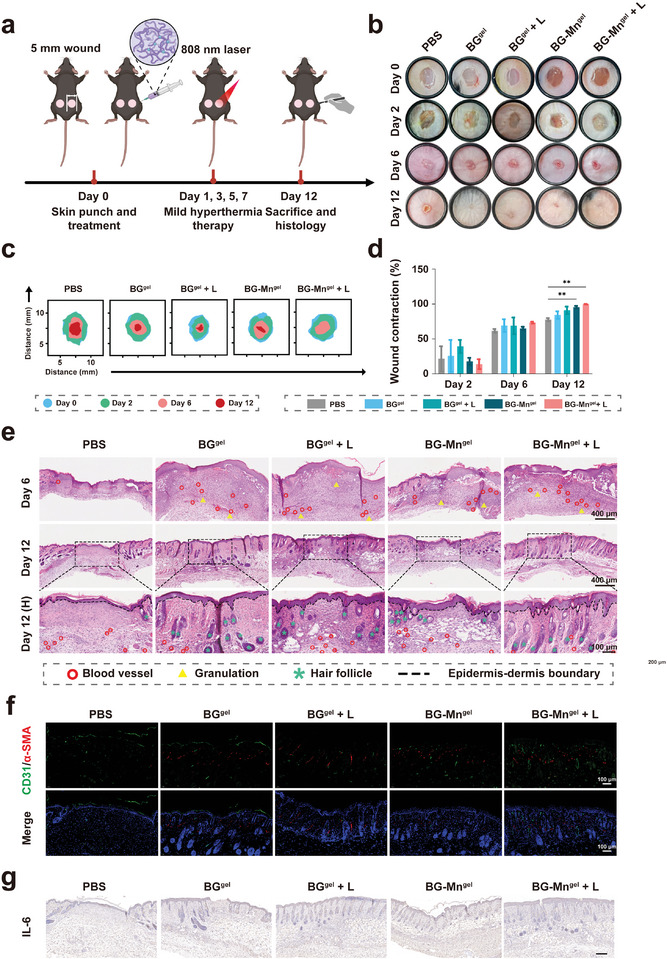
BG‐Mn^gel^ with mild hyperthermia effect promotes wound healing in vivo. a) Schematic showing the experimental procedure for the wound healing evaluation. b) The representative photographs of the wound healing process in C57BL/6 mice with different treatments. c) The corresponding quantitative analysis of wound contraction rate. d) Traces of wound‐bed closure at different time points after various treatments. e) H&E staining images of wound regeneration of different treatment groups. (Red circles mark blood vessel; yellow triangles mark granulation; green asterisks mark hair follicles; dotted lines mark epidermis‐dermis boundary). f) Immunofluorescence staining of CD31/a‐SMA in wounds on day 12. g) Immunohistochemical staining of IL‐6 in tissue sections of wounds from different treatment groups. Data represent mean ± standard deviation (SD) from *n* independent experiments (*n* = 5). Statistical significances between every two groups were calculated via one‐way ANOVA. ^**^ *p* < 0.01, ^***^ *p* < 0.001, ^****^ *p* < 0.0001.

After surgical treatment of melanoma, full‐thickness skin defects and open wounds are often present.^[^ ^35^ ^]^ Thus, Therefore, it is imperative to promote wound closure and healing following tumor therapy. Accordingly, we further evaluated the skin regeneration by constructing a full‐thickness skin defect model in C57BL/6 mice (**Figure** **6**a).

To monitor the progression of wound healing, we quantitatively assessed the healing process in wound dimensions over a 12‐day period. Optical photographs (Figure 6b) and simulated images of wound size and morphology at 4 time points (Figure 6c) demonstrated that the wound healing in the BG‐Mn^gel^ + L group was significantly faster than in the other groups. Although there was no obvious difference at day 2, with an increase in the number of mild heating treatments, the wound contraction rate in the BG‐Mn^gel^ + L group significantly increased from day 6 (Figure 6d). Remarkably, BG‐Mn^gel^ + L group exhibited the highest wound closure rates (99.64%), indicating the promising effect of BG‐Mn^gel^ combined with mild hyperthermia in wound healing rate. This may be attributed to the accelerated angiogenesis and recruitment and migration of fibroblasts facilitated by the release of bioactive ions from BG.^[^ ^36^ ^]^

To further validate this, we conducted a histological examination of the full‐thickness wound recovery employing H&E staining (Figure 6e). The BG‐Mn^gel^ + L group displayed more granulation tissue at day 6, and thicker granulation tissue in this group also exhibited a pronounced wound healing effect at day 12. Surprisingly, we observed more skin appendages, such as hair follicles, in the BG‐Mn^gel^ + L group, which were 1.16 and 1.25 times higher than those in the BG^gel^ and BG^gel^ + L control groups, respectively (Figure S17, Supporting Information). Furthermore, at day 14, the semi‐quantitative epidermal thickness in the BG‐Mn^gel^ + L group was 1.45 times higher than that in the BG^gel^ group (Figure S18, Supporting Information), further indicating the superior wound repair effect of BG‐Mn^gel^ + L group.

In addition, angiogenesis plays a pivotal role in wound healing by facilitating the supply of nutrients and oxygen to cells throughout the tissue repair process.^[^ ^37^ ^]^ To visually observe neovascularization in the tissues of each group, we performed immunofluorescent staining using CD31 and α‐SMA. As shown in Figure 6f, the expression levels of CD31 and α‐SMA in the BG‐Mn^gel^ + L treated tissues were elevated compared to the control group, indicating a significant enhancement in both blood vessel formation and maturation. The semi‐quantitative analysis of vascular density further corroborates these results, revealing a marked augmentation in the vascular network integral to efficacious tissue repair and regeneration (Figure S19, Supporting Information).

In the wound repair process, the transition from a pro‐inflammatory to a reparative microenvironment must be tightly regulated. Interleukin (IL)−6 plays a pivotal role in cutaneous wound healing.^[^ ^38^ ^]^ Hence, we conducted immunohistochemical analysis to examine the expression of IL‐6 during the wound repair process. As illustrated in Figure 6g and Figure S20 (Supporting Information), there was a significant downregulation of IL‐6 expression in the BG‐Mn^gel^ + L group, indicating that the composite hydrogel promotes the transition from an inflammatory to a reparative microenvironment, facilitating wound repair.

## 3 Conclusion

The current treatment landscape for malignant melanoma requires a therapeutic platform that can achieve rapid eradication of in situ tumor tissue, treat residual tumor sites, prevent recurrence, and address the wound repair challenges after surgical tumor resection. Injectable hydrogels, with their unique non‐invasiveness, tunability, and multifunctionality, have been widely employed in cancer therapy to achieve long‐term retention and sustained release of drugs. In this study, we successfully developed a bioactive injectable hydrogel composed of Mn‐doped bioactive glass (BG) and sodium alginate as a versatile platform for in situ treatment of malignant melanoma, aiming to induce enhanced anti‐tumor immune response and promote wound healing. The BG‐Mn^gel^ exhibited excellent photothermal responsiveness and photothermal stability, enabling convenient mild hyperthermia treatments at the tumor site. We validated the combined treatment mode of BG‐Mn^gel^ + L and α‐PD‐1 in a B16F10 subcutaneous tumor model, which significantly enhanced the anti‐tumor efficacy in vivo and accompanied by an enhanced anti‐tumor immune memory response. Additionally, the angiogenic and tissue repair‐enhancing capabilities of BG‐Mn^gel^, along with the release of various active ions such as SiO_4_^4−^, Ca^2+^, and Mn^2+^ under mild hyperthermia conditions, facilitated the rapid healing of postoperative wounds. Overall, this in situ bioactive hydrogel holds great promise for the treatment of malignant melanoma.

## Conflict of Interest

The authors declare no conflict of interest.

## Supporting information

Supporting Information

## Data Availability

The data that support the findings of this study are available from the corresponding author upon reasonable request.
