# Supplementary material for: A Bioactive Injectable Hydrogel Regulates Tumor Metastasis and Wound Healing for Melanoma via NIR‐Light Triggered Hyperthermia
Source: Adv Sci (Weinh). 2024 May 5;11(26):2402208. doi: 10.1002/advs.202402208 (PMC11234446; doi:10.1002/advs.202402208)
Supplement: Supplementary file 1 — Supporting Information [file ADVS-11-2402208-s001.pdf]

ADVANCED SCIENCE
Open Access

Supporting Information

for *Adv. Sci.*, DOI 10.1002/advs.202402208

A Bioactive Injectable Hydrogel Regulates Tumor Metastasis and Wound Healing for Melanoma via NIR-Light Triggered Hyperthermia

*Xueyi Liu, Meifang Shen, Tiejun Bing, Xinyun Zhang, Yifan Li, Qing Cai, Xiaoping Yang and Yingjie Yu**

Supporting Information

A Bioactive Injectable Hydrogel Regulates Tumor Metastasis and Wound Healing for Melanoma *via* NIR-Light Triggered Hyperthermia

*Xueyi Liu, Meifang Shen, Tiejun Bing, Xinyun Zhang, Yifan Li, Qing Cai, Xiaoping Yang, Yingjie Yu**

X. Liu, M. Shen, Y. Li, Q. Cai, X. Yang, Y. Yu

State Key Laboratory of Organic-Inorganic Composites; Beijing Laboratory of Biomedical Materials; College of Life Science and Technology

Beijing University of Chemical Technology

Beijing 100029, China

Email: yuyingjie@mail.buct.edu.cn

T. Bing, X. Zhang

Immunology and Oncology center

ICE Bioscience

Beijing 100176, China

SUPPLEMENTAL MATERIALS AND METHODS

Materials

Ethyl orthosilicate (TEOS) and triethyl phosphate (TEP) were purchases from Aladdin. Calcium nitrate tetrahydrate was purchased from Beijing Tongguang Fine Chemical *Co., Ltd.* (China). Manganous acetate was purchased from Sinopharm Chemical Reagent Co., *Ltd* (China). sodium alginate (SA) (viscosity 200 ± 20 mPa·s) was purchased from Shanghai Macklin Biochemical *Co., Ltd.* (China). Dextronic acid δ-lactone (GDL) was purchased from Shanghai Aladdin *Co., Ltd.* (China).

Cell lines and animals

B16F10 and L929 cells were used in the in vitro experiments. B16F10 cells were cultured in DMEM media. L929 cells were cultured in MEM media. Culture medium were supplemented with 10% (v/v) FBS and 1% (v/v) P/S. The cell lines were cultured in an incubator at 37°C containing 5% (v/v) CO_2. Female C57BL/6 mice were purchased from SPF Biotechnology (Beijing, China).

Synthesis and characterization of BG and BG-Mn

Firstly, Si-Ca-P-based bioactive glass nanoparticles (BG) and Mn-doped bioactive glass nanoparticles (BG-Mn) with spherical morphology were synthesized using a typical sol-gel method. BG and BG-Mn were prepared with the compositions being set as 60 SiO_2 : 36 CaO : 4 P_2O_5 and 60 SiO_2 : 31 CaO : 5 MnO : 4 P_2O_5 in mol%. The morphological characteristics and distribution of the nanoparticles were observed by scanning electron microscope (SEM, SU8020, Japan) and analyzed by

dynamic light scattering (DLS), respectively. The elemental compositions of BG and BG-Mn were analyzed using fourier transform infrared spectroscopy (FTIR, Nicolet 8700, Nicolet) and X-ray photoelectron energy (XPS, ESCALAB 250, THERMO VG). The absorption of nanoparticles and hydrogels was exhibited by ultraviolet-visible spectroscopy (UV-vis, Shimadzu, Japan). The near-infrared (NIR) laser was employed for the experiment with irradiation operation. The temperature and thermal images of the solution were recorded by a thermal imaging camera.

Photothermal conversion efficiency (η) of BG-Mn

To obtain the photothermal conversion efficiency (η) for BG-Mn. The BG-Mn were dissolved in water at concentration of 2 mg·mL^{-1}, which is consistent with concentration of the BG-Mn nanoparticles in the SA pre-gel solution. Then the BG-Mn dispersion was irradiated by 808 nm laser (1 W·cm^{-2}) for 10 min to achieve the maximum plateau of temperature, and the laser was turned off for natural cooling to room temperature. The heating and cooling curves were monitored. Thus, η can be calculated referring to the following Equation (1):

$$\eta = \frac{\boldsymbol{hs(T_{max} - T_{surr}) - Q_{diss}}}{\boldsymbol{I(1 - 10^{A_\lambda})}} \quad \text{..........................(1)}$$

Where h is the heat transfer coefficient, S is the surface area of the used holder, T_{max} and T_{surr} denote the maximum steady-state temperature and room temperature of the ambient environment, Q_{Diss} is the heat wastage from the light loss of the solvent and holder, I is the laser intensity (1 W·cm^{-2}), and A^{λ} represents the absorbance of corresponding BG-Mn dispersion at 808 nm. hS can be calculated referring to the following Equation (2):

$$\tau_s = \frac{\boldsymbol{mC_p}}{\boldsymbol{hs}} \quad \text{....................................(2)}$$

Where τ_s is the time constant, m is the mass of the solution, and C_p is the heat capacity of corresponding solvent. Finally, the PCE can be calculated from the

method above.

Synthesis and characterization of BG containing hydrogel

BG^{gel} and $BG\text{-}Mn^{gel}$ were fabricated through a facile mixing method as sodium alginate (SA) could rapidly form hydrogel in the presence of Ca^{2+} and Mn^{2+} ions released from BG and BG-Mn. In brief, 3 % wt SA solution and BG or BG-Mn aqueous solution were homogeneous mixed under ultrasound probe. With the addition of a certain amount of GDL powders, the gelation of BG^{gel} and $BG\text{-}Mn^{gel}$ immediately started with the Ca^{2+} and Mn^{2+} ions released from the BG and BG-Mn. The rheological property of BG^{gel} and $BG\text{-}Mn^{gel}$ was characterized by a hybrid rheometer (Discovery HR-1, TA, USA). In the measurement, the samples were placed between the parallel plates (20 mm in diameter) with a gap of 1000 μm. Scanning electron microscope (SEM, S4800, Hitachi, Japan) was employed to observe the morphology of BG^{gel} and $BG\text{-}Mn^{gel}$. Elemental mapping images were obtained under the same parameters, together with energy dispersive spectroscopy (EDS, Inca X-Max, UK) to evaluate elemental compositions.

In vitro and *in vivo* photothermal performance of $BG\text{-}Mn^{gel}$

To study the photothermal properties of $BG\text{-}Mn^{gel}$, the as-prepared hydrogel was placed into the 20 mm plates and then exposed to an 808 nm NIR laser at a power density of 1 $W{\cdot}cm^{-2}$ for 10 min. The temperature variation was recorded by thermal imaging camera.

In vivo infrared thermal imaging was established as described above. The tumor was grown by inoculating 100 μL of B16F10 cells with a concentration of 1×10^6 cells in PBS into the right flank of each C57BL/6 mouse (Female, 6 weeks old). After the tumor volume reached about 50 mm^3, 100 μL of BG^{gel} and $BG\text{-}Mn^{gel}$ was injected

directly into the tumor area. Subsequently, the tumor region was then irradiated with an 808 nm laser for 10 min.

Release properties of hydrogel

The BG-Mn^{gel} was incubated in 3 mL of PBS (pH 5.5 or pH 7.4). At the desired interval, supernatants were all retrieved and 3 mL fresh solution was replenished to continue the release test. Each sample of the light group was irradiated with an 808 nm laser at an appropriate power density for 10 min at 4 h and 48 h after sampling. The supernatants were collected and submitted to inductively coupled plasma optical emission spectrometer (ICP-OES, ICPS-7500, Shimadzu, Japan) to detect the release amounts of various elements.

Evaluation of *in vitro* cytocompatibility

Normal mouse fibroblast (L929) was used to evaluate the cytotoxicity of BG^{gel} and BG-Mn^{gel}. Cell counting kit-8 (CCK-8, Beyotime, China) was used to detect cell viability. The optical density value was read at 450 nm using microplate reader. Furthermore, calcein-AM/PI (Aladdin, China) fluorescence staining assay was applied to detect living and dead cells. The outcomes were observed with a confocal laser scanning microscope (CLSM, TCS SP8, Leica, Germany).

The effect of BG^{gel} and BG-Mn^{gel} on promoting tissue regeneration was evaluated by cell scratch assay. L929 cells were seeded on 24-well plates. When cell fusion degree was up to 100%, the scratch was made by 200 μL pipette tips. After rinsing with PBS for 2 times, the scratch area was photographed by inverted fluorescence microscope. BG^{gel} and BG-Mn^{gel} were added into the corresponding well to co-cultivate with L929 cells. After incubation for 24 h, the scratch area was photographed again to record cells migration effect from peripheral area. The blank group was the cells incubated with the serum-free medium without the presence of the hydrogels. The cells migration rate was calculated by the following equation. S_0 was

cells area in scratch area at 0 h, S_t was cells area in scratch area at 24 h:

The cells migration area rate = $(M_t-M_0)/M_0 \times 100\%$

In vitro cellular uptake of Mn^{2+} released from BG-Mn^{gel}

B16F10 cells were placed into 6-well plates (5×10^6 cells/well) and allowed to adhere overnight. The cells were then co-cultured with BG-Mn^{gel} for 1, 4 and 7 h. The light group was exposed to an 808 nm laser for keeping the temperature at 43°C. The cells were then washed three times with cold PBS. Subsequently, 500 μL of concentrated HNO_3 and 500 μL of 30% H_2O_2 solution were added into each well. After 4 h digestion, the mixture was collected for atomic absorption spectrometry (AAS) test.

Evaluation of STING pathway activation.

In vitro fluorescence staining

B16F10 cells were treated with BG^{gel} and BG-Mn^{gel} for 24 h, and PBS served as control treatment. The light group was exposed to an 808 nm laser for keeping the temperature at 43°C. The above cells were washed with PBS and further incubated with Phospho-STING (Ser366) (D1C4T) Rabbit mAb (Cell Signaling Technology, USA) for 2 h at 37°C. Subsequently, the cells were incubated with Goat Anti-Rabbit IgG H&L (Alexa Fluor® 488) for 1 h at 37°C. The expression of p-STING inside the cells was observed by CLSM.

Western blot

B16F10 cells were seeded in 6-well plates (1×10^6cells/well) and allowed to adhere overnight. The cells with same treatments as aforementioned were collected after 6 h incubation and washed three times with cold PBS, then maintained in

medium for 48 h. RIPA lysis buffer with protease and phosphatase inhibitors was added into well. The proteins of cells were extracted through centrifuge at a speed of 12000 rpm for 5 min. Protein content quantification was carried out by the BCA protein assay kit. Then, the electrophoreses process was conducted through SDS-PAGE by a gel-electrophoretic apparatus (Bio-Rad mini, USA), and the proteins were transferred to the PVDF films and incubated with the antibodies against various proteins overnight on a shaker at 4°C. Subsequently, the PVDF films were washed 5 times and incubated with HRP conjugated antibodies for 1 h. The Western blot images were obtained by Amersham Imager 600 (AI600, General Electric *Co., Ltd.,* USA) with 300 μL of ECL chemiluminescent reagent (Beyotime biotechnology *Co., Ltd.,* P0018AS) added on the top of the membrane.

Immune response analysis *in vitro*

To investigate the maturation of dendritic cells (DCs) *in vitro*, the bone marrow is extracted from the femur and tibia of 6-week-old C57BL/6 mice. After collection, the BMDCs are flushed our using a syringe filled with culture medium. Red blood cells are lysed using a red blood cell lysis buffer, leaving behind a mononuclear cell population. The mononuclear cells are cultured in RPMI 1640 medium supplement with 10% FBS, GM-CSF (20 ng/mL) and IL-4 (10 ng/mL) at 37°C with 5% CO_2. Fresh medium supplemented with GM-CSF is added every two to three days to support cell growth and differentiation.

B16F10 cells were seeded on 6-well plates (1×10^6 cells/well) with hydrogels as aforementioned. Afterwards, the treated B16F10 cells were co-cultured with the obtained BMDCs for 24 h.

Then, the maturation of BMDCs is assessed by analyzing the expression of surface markers such as CD11c, CD80 and CD86. The BMDCs were stained with the corresponding antibodies (PE anti-mouse CD11c antibody, FITC anti-mouse CD80 antibody, APC anti-mouse CD86 antibody, Bio legend, USA) for 1 h. The maturation

of BMDCs was assessed by flow cytometry.

Establishment of B16F10 subcutaneous tumor model

To establish subcutaneous tumor model, B16F10 cells (3×10^6 cells/well) were dispersed in PBS buffer and implanted into right flank of C57BL/6 mice.

Immune response analysis *in vivo*

When the size of tumor reached 100 mm^3, the mice bearing B16F10 subcutaneous tumor were divided into five groups randomly and administrated with PBS, BG^{gel}, BG^{gel} + L, $BG\text{-}Mn^{gel}$ and $BG\text{-}Mn^{gel}$ + L through intratumor injection. In addition, each mouse in the light group was irradiated with an 808 nm laser for 3 times post injection. The tumor volume was monitored at a time interval of 2 days. Tumor volume was calculated by the following formula: Volume = (Length × $Width^2$)/2

To evaluate immune response *in vivo*, the tumor and lymph were harvested after various treatments. To investigate the DCs maturation *in vivo*, the lymph from each treatment were collected and homogenized into single cell suspension. The cell suspensions were stained with primary antibodies of PE anti-mouse CD11c antibody, FITC anti-mouse CD80 antibody, and APC anti-mouse CD86 antibody for flow cytometry analysis. The matured DCs were denoted as $CD11c^+CD80^+CD86^+$ cells.

To investigate the infiltration of antitumor T cells, the tumor tissues were cut into small pieces and homogenized into single cell suspension. To determine the tumor infiltration of $CD8^+$ T cells, the isolated cell suspensions were incubated with PE anti-mouse CD3 antibody, FITC anti-mouse CD8 antibody, and APC anti-mouse CD4 antibody. The $CD8^+$ T cells were marked as $CD3^+CD8^+$ T cells, which was presented as the percentage of $CD8^+$ T cells in $CD3^+$ T cells.

To detect the ability in converting M2 to M1 polarization of macrophages, the tumor tissues were cut into small pieces and homogenized into single cell suspension.

Then the isolated cell suspensions were incubated with PE anti-mouse F4/80 antibody, APC anti-mouse CD206 antibody and FITC anti-mouse CD80 antibody. The cell population of M2-type macrophages (characterized as $F4/80^{+}CD80^{-}CD206^{+}$ cells) and M1-type macrophages (characterized as $F4/80^{+}CD80^{+}CD206^{-}$ cells) were determined using flow cytometry analysis. Then, the M1/M2 ratio was calculated to assess their ability to restore the antitumor activities.

To examine, the tumor tissues were cut into small pieces and homogenized into single cell suspension. Then the isolated cell suspension were incubated with APC anti-mouse CD45 antibody, PE anti-mouse CD11b antibody and FITC anti-mouse GR-1 antibody. The MDSCs (characterized as $CD11b^{+}GR\text{-}1^{+}CD45^{+}$ cells) were determined using flow cytometry analysis.

Immune memory effect of combination therapy *in vivo*

To evaluate the effects of long-term immunization, the spleens were cut into small pieces and homogenized into single cell suspension. Then the isolated cell suspensions were incubated with PE anti-mouse CD3 antibody, FITC anti-mouse CD8 antibody, APC anti-mouse CD62L antibody and PerCP/Cyanine5.5 anti-mouse CD44 antibody. The central memory T cells (characterized as $CD3^{+}CD8^{+}CD44^{+}CD62L^{+}$ cells) were determined using flow cytometry analysis.

Wound healing performance *in vivo*

The effects of BG^{gel} and $BG\text{-}Mn^{gel}$ on wound healing was evaluated on a full-thickness mice wound model. In brief, the C57BL/6 mice (Female, 6-week old) were anesthetized and their backs were shaved. A round full-thickness cutaneous wound (5 mm × 5 mm) area was created on the back. The mice were randomly divided into 5 groups and administrated with PBS, BG^{gel}, BG^{gel} + L, $BG\text{-}Mn^{gel}$, and

BG-Mn^{gel} + L. To observe the wound healing process, wounds were measured using a plastic ring and photographed at day 0, 2, 6, and 12. Wound contraction rates were calculated as follows:

$$\textit{Wound contraction rate} = (\textit{Wound area at Day0} - \textit{Wound area at certain day}) / \textit{Wound area at Day0} \times 100\ \%$$

The harvested wound tissues were further evaluated using the histological analysis. Briefly, the wound site and surrounding skin were collected at Day 2, 6, and 12 that was fixed, embedded and sectioned for hematoxylin-eosin (H&E) stain. Then the slices were observed by microscope (Nikon Eclipse E100, Japan).

Statistical analysis

GraphPad Prism 9.5 (GraphPad, La Jolla, CA, USA) is used for statistical analysis. The results are shown as the mean ± SD analyzed using the ordinary one-way ANOVA and two-way ANOVA. *$p < 0.05$; **$p < 0.01$; ***$p < 0.001$, **** $p < 0.0001$.

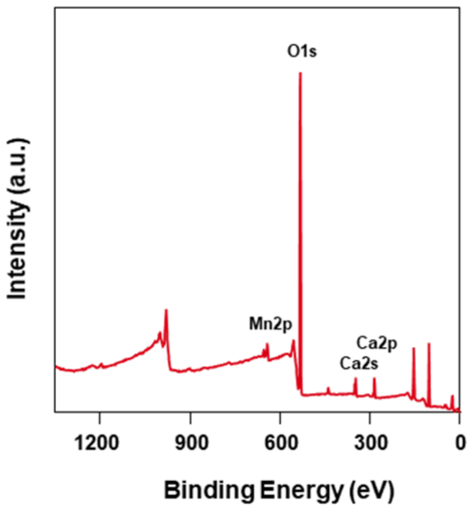

Figure S1. XPS spectra of BG-Mn.

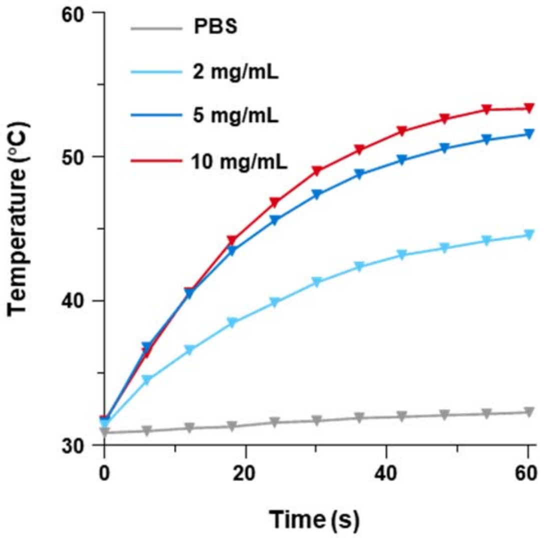

Figure S2. Temperature profiles of different concentrations of BG-Mn suspension under irradiation of 808 nm laser.

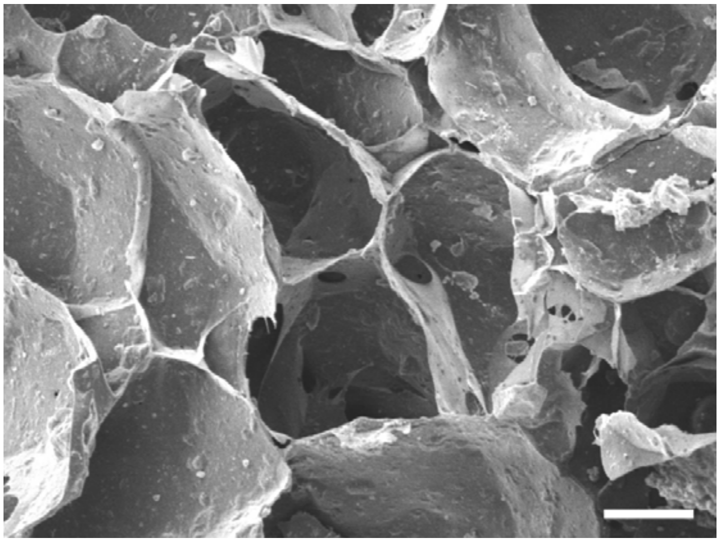

Figure S3. SEM image of BG^{gel}. Scale bar = 100 μm.

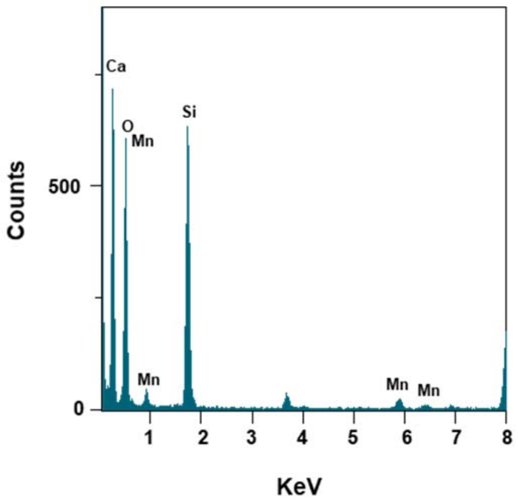

Figure S4. EDS analysis of BG-Mn^{gel}.

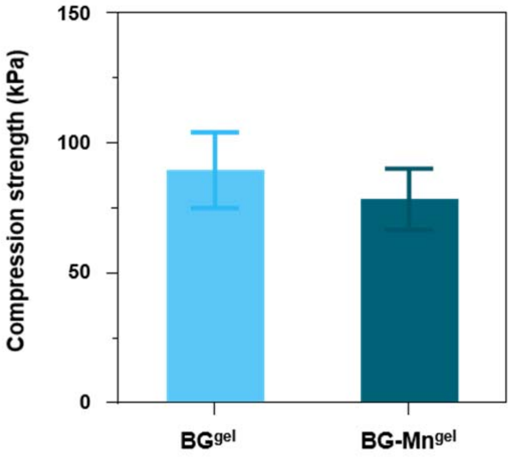

Figure S5. The compression strength of BG^{gel} and $BG\text{-}Mn^{gel}$.

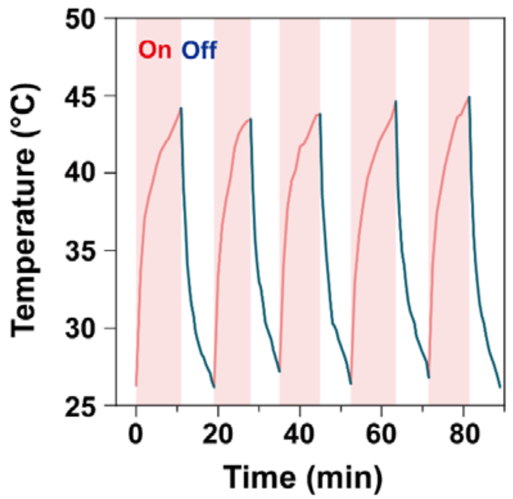

Figure S6. Evaluation for photostability of BG-Mn^{gel} by monitoring the temperature elevation for five on/off laser irradiation cycles.

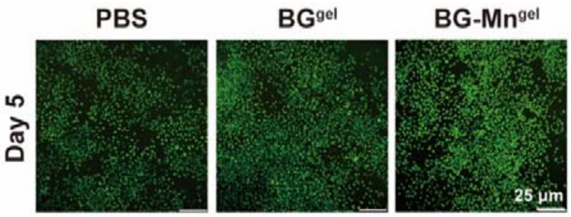

Figure S7. CLSM images showing live & dead staining of L929 cells after co-cultivation with BG^{gel} and BG-Mn^{gel} for day 5.

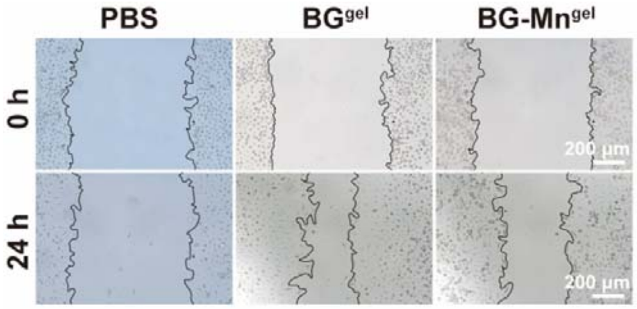

Figure S8. L929 cells migrating from the peripheral area into the scratch area after co-cultivation with BG^{gel} and $BG\text{-}Mn^{gel}$ for 24 h.

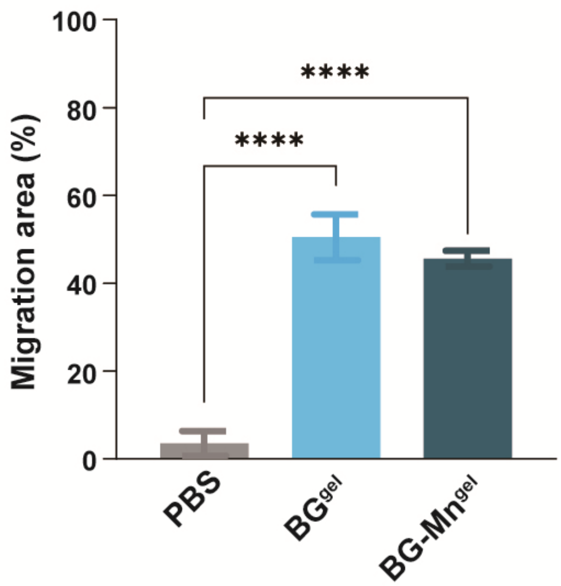

Figure S9. The quantitative data of L929 cells migration rate (n = 3).

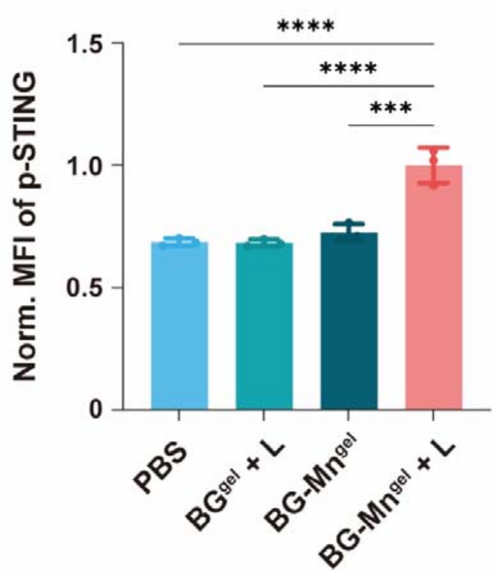

Figure S10. Quantification of STING activation based on the fluorescence intensity in the CLSM images shown in Figure 2c. Mean fluorescence intensity of p-STING.

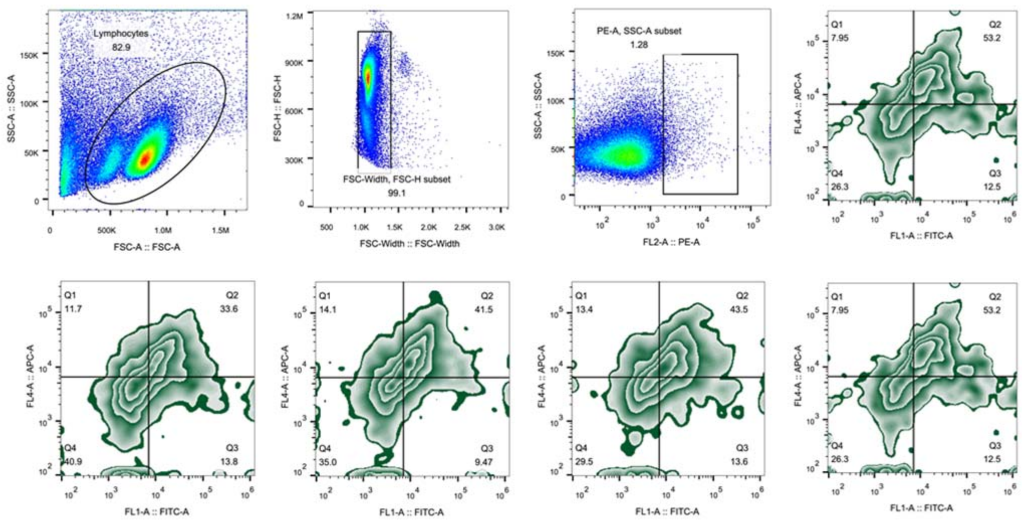

Figure S11. The gating strategy of DCs maturation in TDLNs from B16F10 tumor bearing mice with different treatments.

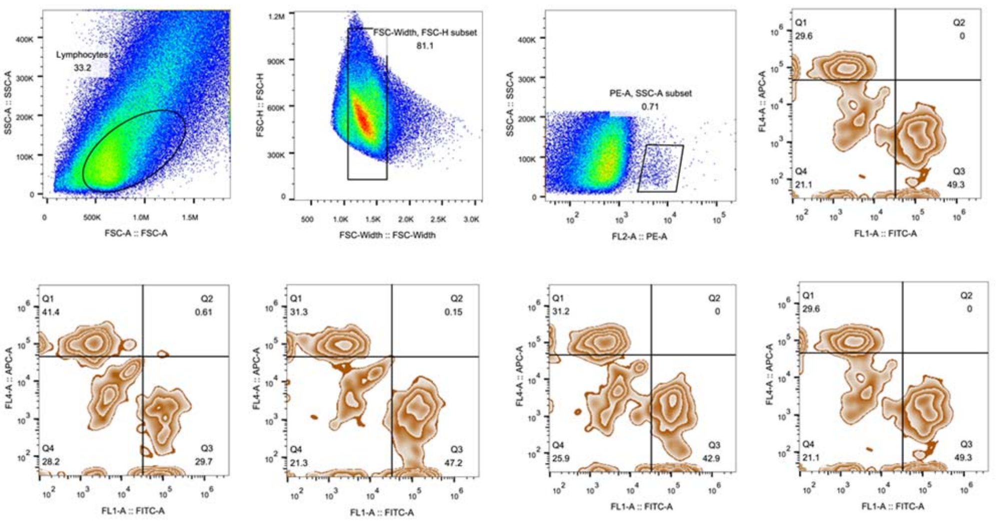

Figure S12. The gating strategy of CD3^{+}CD8^{+} T cells in tumor sites extracted from B16F10 tumor bearing mice with different treatments.

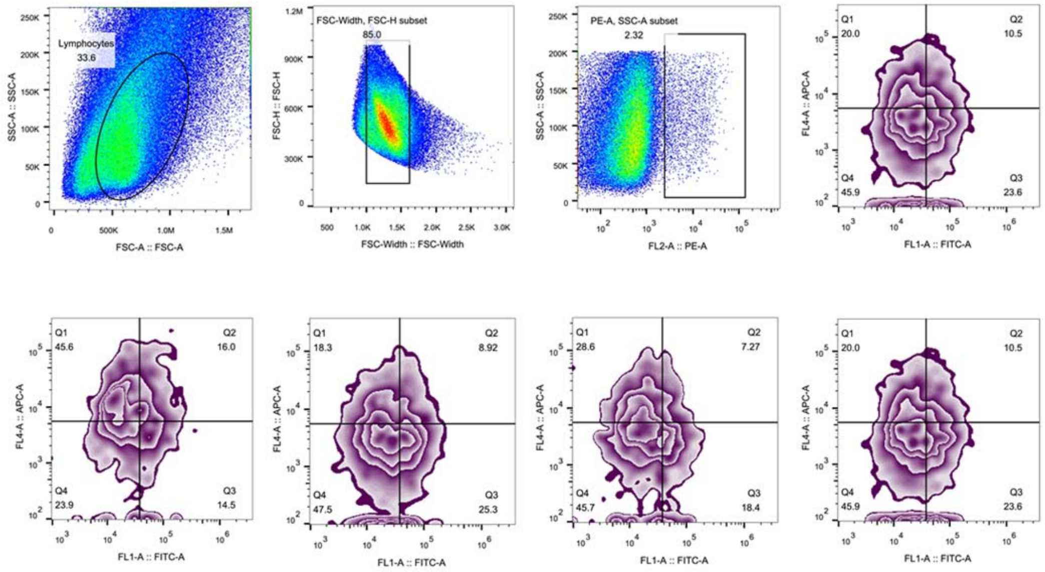

Figure S13. The gating strategies and representative flow cytometry images of M1 macrophages ($CD80^{+}CD206^{-}$) and M2 macrophages ($CD80^{-}CD206^{+}$) within tumor tissues after various treatments.

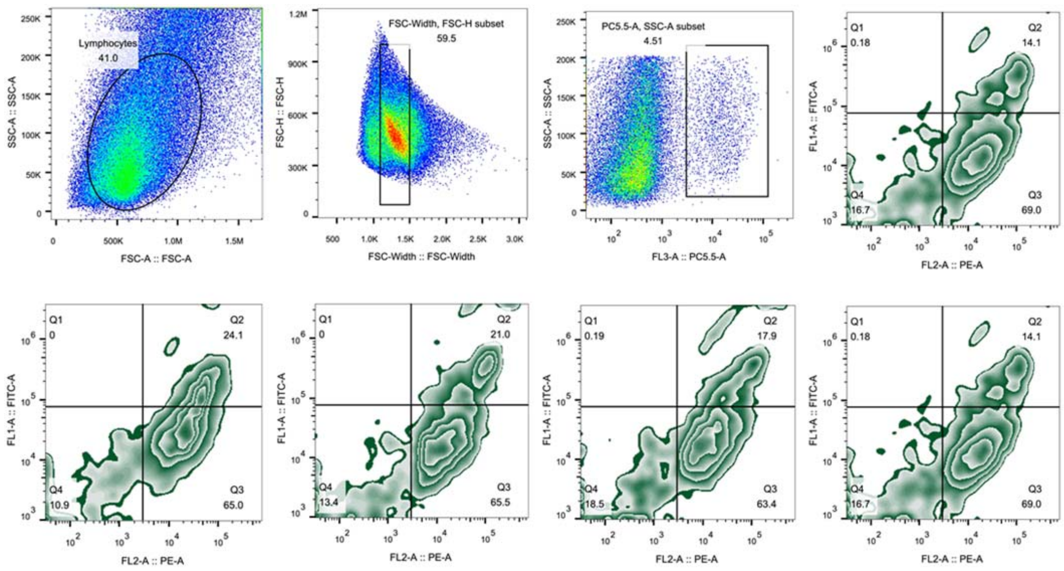

Figure S14. The gating strategy of CD11b$^+$GR-1$^+$CD45$^+$ cells in tumor sites from B16F10 tumor bearing mice with different treatments.

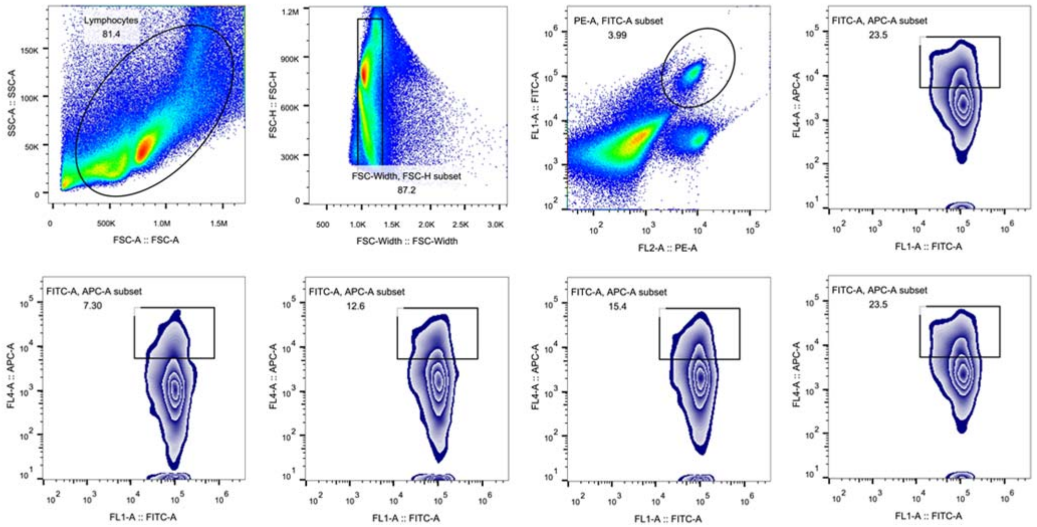

Figure S15. The gating strategy and typical flow cytometer analysis profiles of $CD3^{+}CD8^{+}$ T cells in spleens from B16F10 tumor bearing mice with various treatments.

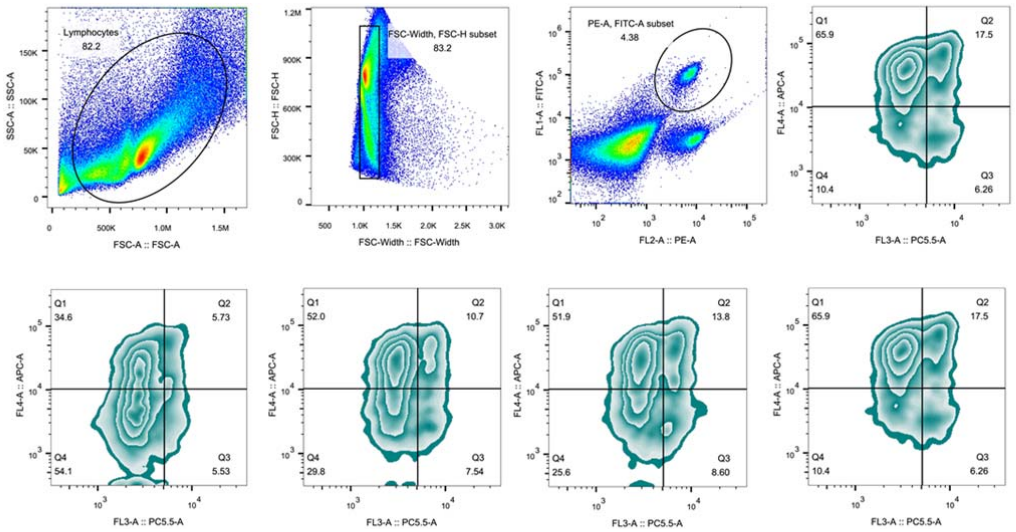

Figure S16. The gating strategy and typical flow cytometer analysis profiles of $CD3^+CD8^+CD44^+CD62L^+$ cells in spleens from B16F10 tumor bearing mice with various treatments.

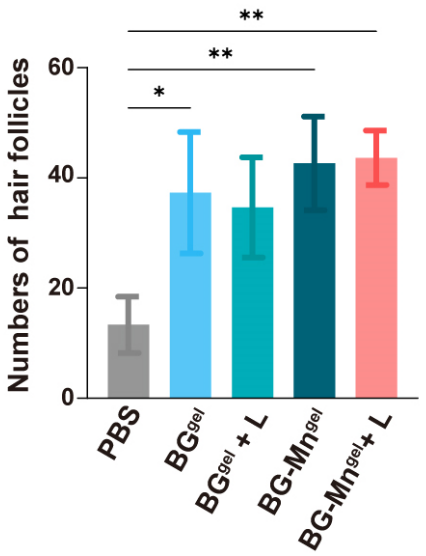

Figure S17. Quantitative analysis of the hair follicles on day 12.

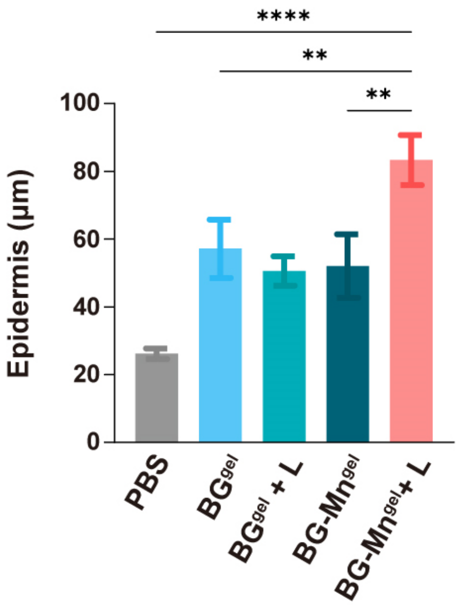

Figure S18. Quantitative analysis of the epidermis thickness on day 12.

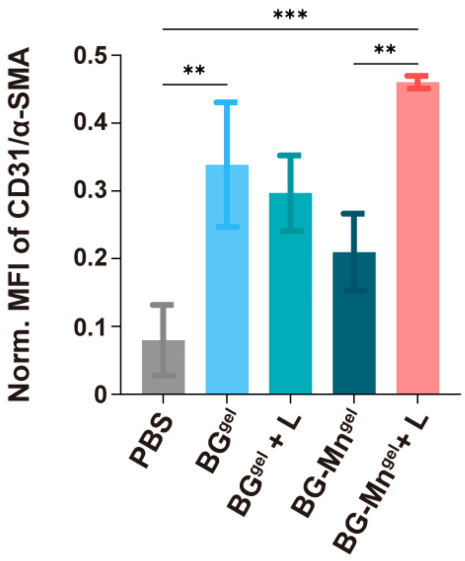

Figure S19. The corresponding semi-quantitative analysis of the according vascular density.

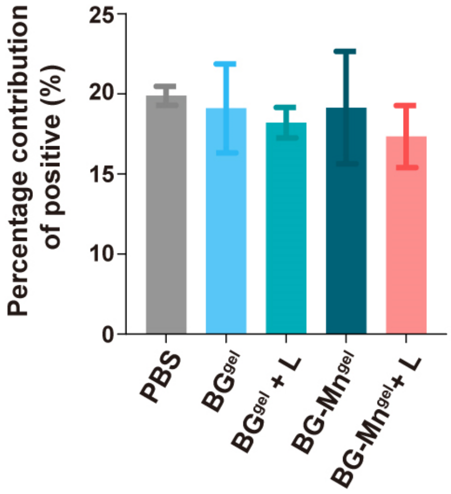

Figure S20. The corresponding semi-quantitative analysis of percentage contribution of positive on day 12.
